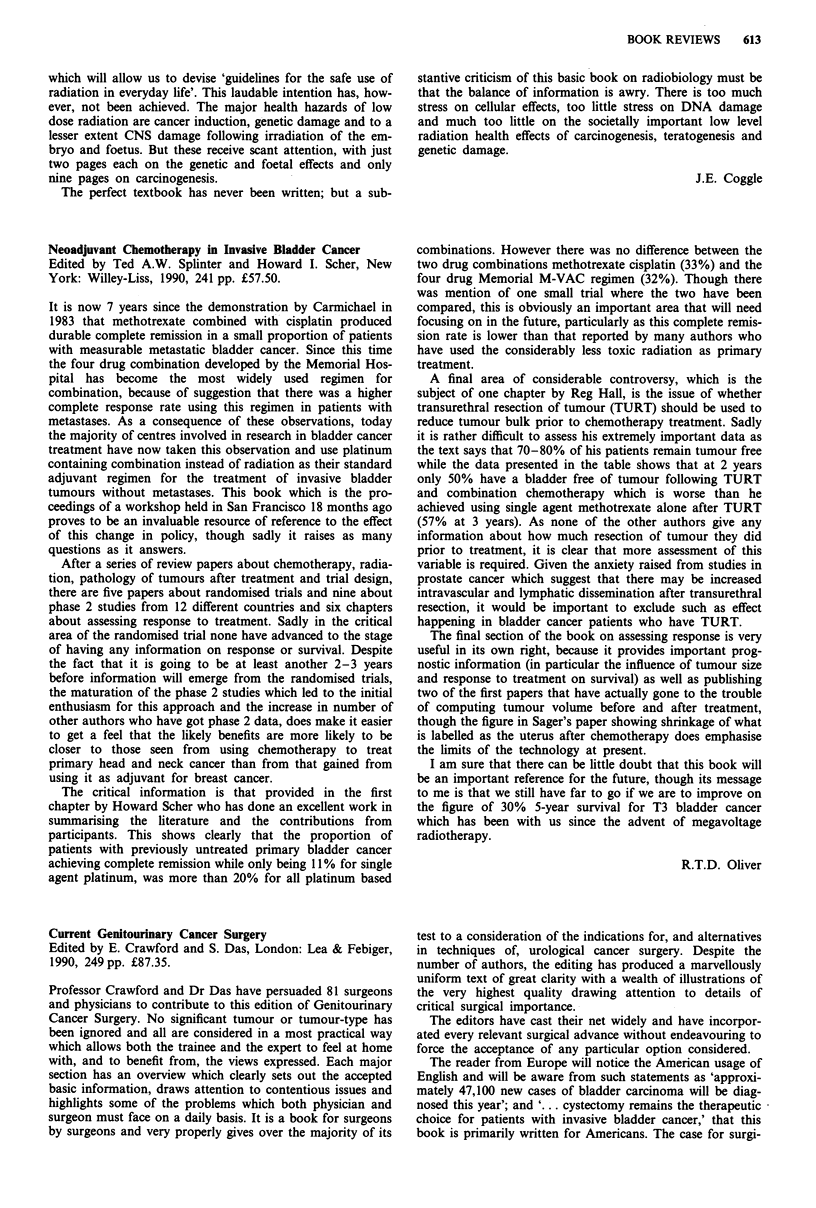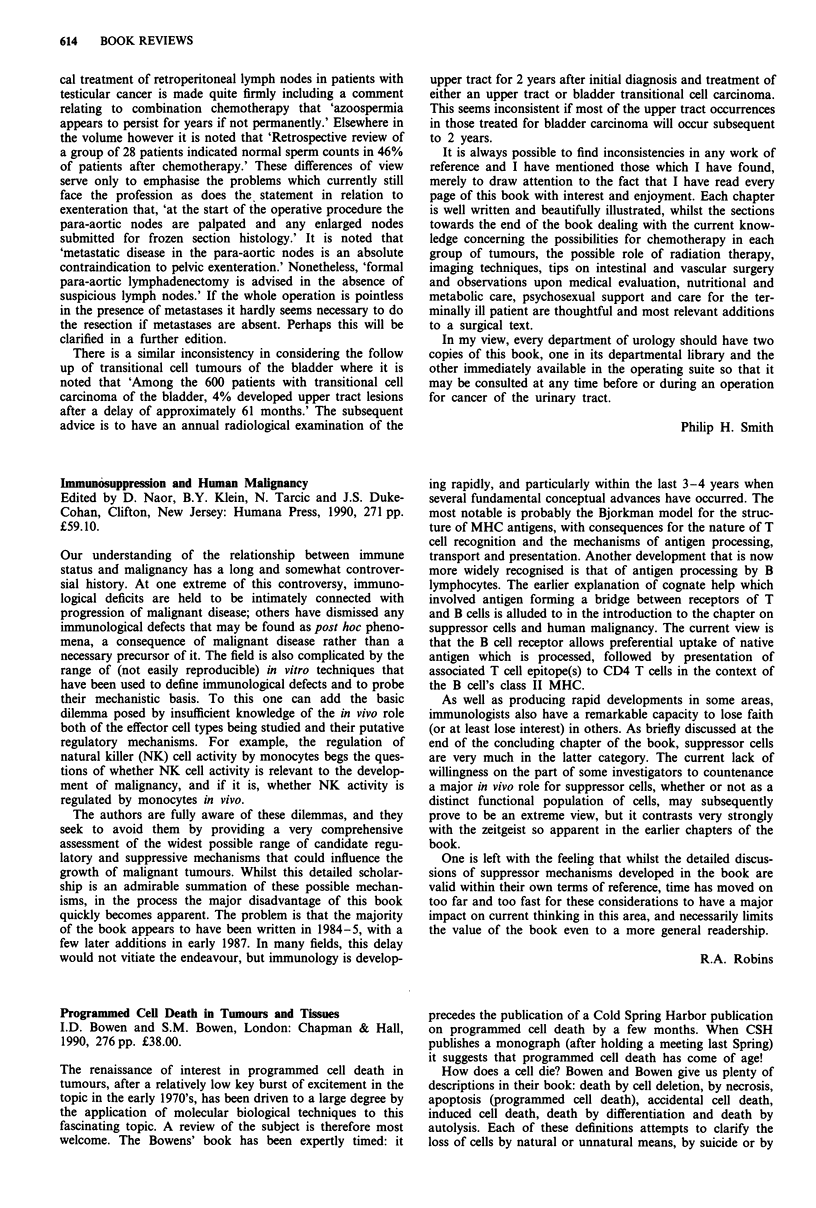# Current Genitourinary Cancer Surgery

**Published:** 1991-09

**Authors:** Philip H. Smith


**Current Genitourinary Cancer Surgery**
Edited by E. Crawford and S. Das, London: Lea & Febiger, 1990, 249 pp. £87.35.

Professor Crawford and Dr Das have persuaded 81 surgeons and physicians to contribute to this edition of Genitourinary Cancer Surgery. No significant tumour or tumour-type has been ignored and all are considered in a most practical way which allows both the trainee and the expert to feel at home with, and to benefit from, the views expressed. Each major section has an overview which clearly sets out the accepted basic information, draws attention to contentious issues and highlights some of the problems which both physician and surgeon must face on a daily basis. It is a book for surgeons by surgeons and very properly gives over the majority of its test to a consideration of the indications for, and alternatives in techniques of, urological cancer surgery. Despite the number of authors, the editing has produced a marvellously uniform text of great clarity with a wealth of illustrations of the very highest quality drawing attention to details of critical surgical importance.

The editors have cast their net widely and have incorporated every relevant surgical advance without endeavouring to force the acceptance of any particular option considered.

The reader from Europe will notice the American usage of English and will be aware from such statements as 'approximately 47,100 new cases of bladder carcinoma will be diagnosed this year'; and '. . . cystectomy remains the therapeutic choice for patients with invasive bladder cancer,' that this book is primarily written for Americans. The case for surgical treatment of retroperitoneal lymph nodes in patients with testicular cancer is made quite firmly including a comment relating to combination chemotherapy that 'azoospermia appears to persist for years if not permanently.' Elsewhere in the volume however it is noted that 'Retrospective review of a group of 28 patients indicated normal sperm counts in 46% of patients after chemotherapy.' These differences of view serve only to emphasise the problems which currently still face the profession as does the statement in relation to exenteration that, 'at the start of the operative procedure the para-aortic nodes are palpated and any enlarged nodes submitted for frozen section histology.' It is noted that 'metastatic disease in the para-aortic nodes is an absolute contraindication to pelvic exenteration.' Nonetheless, 'formal para-aortic lymphadenectomy is advised in the absence of suspicious lymph nodes.' If the whole operation is pointless in the presence of metastases it hardly seems necessary to do the resection if metastases are absent. Perhaps this will be clarified in a further edition.

There is a similar inconsistency in considering the follow up of transitional cell tumours of the bladder where it is noted that 'Among the 600 patients with transitional cell carcinoma of the bladder, 4% developed upper tract lesions after a delay of approximately 61 months.' The subsequent advice is to have an annual radiological examination of the upper tract for 2 years after initial diagnosis and treatment of either an upper tract or bladder transitional cell carcinoma. This seems inconsistent if most of the upper tract occurrences in those treated for bladder carcinoma will occur subsequent to 2 years.

It is always possible to find inconsistencies in any work of reference and I have mentioned those which I have found, merely to draw attention to the fact that I have read every page of this book with interest and enjoyment. Each chapter is well written and beautifully illustrated, whilst the sections towards the end of the book dealing with the current knowledge concerning the possibilities for chemotherapy in each group of tumours, the possible role of radiation therapy, imaging techniques, tips on intestinal and vascular surgery and observations upon medical evaluation, nutritional and metabolic care, psychosexual support and care for the terminally ill patient are thoughtful and most relevant additions to a surgical text.

In my view, every department of urology should have two copies of this book, one in its departmental library and the other immediately available in the operating suite so that it may be consulted at any time before or during an operation for cancer of the urinary tract.

Philip H. Smith